# Cardiac telerehabilitation under 5G internet of things monitoring: a randomized pilot study

**DOI:** 10.1038/s41598-023-46175-z

**Published:** 2023-11-02

**Authors:** Xiaojie Li, Lvheng Zhao, Tao Xu, Guofeng Shi, Jie Li, Wei Shuai, Yanqun Yang, Yang Yang, Weiyi Tian, Yixia Zhou

**Affiliations:** 1 Nursing School, Guizhou University of Traditional Chinese Medicine, Guiyang, 550025 Guizhou China; 2 Department of Cardiovascular Internal Medicine, Second Affiliated Hospital, Guizhou University of Traditional Chinese Medicine, Guiyang, Guizhou China; 3 Second Affiliated Hospital, Guizhou University of Traditional Chinese Medicine, Guiyang, Guizhou China; 4 Guizhou University of Traditional Chinese Medicine, Guiyang, 550025 Guizhou China

**Keywords:** Cardiology, Diseases, Health care, Health occupations, Medical research

## Abstract

Owing to issues such as time and cost, patients often show poor acceptance of and adherence to center-based cardiac rehabilitation (CBCR), which impacts the effectiveness of rehabilitation. Therefore, there is growing interest in home-based cardiac rehabilitation and cardiac telerehabilitation (CTR), which entail less time and cost than CBCR. This study aimed to compare the changes in physiological and psychological indicators, compliance, and satisfaction after CTR and CBCR. In this single-blind, randomized, controlled trial, the intervention group received CTR via the 5G Internet of Things platform, while the control group received CBCR. Data from 50 patients (age 66.28 ± 4.01 years) with acute myocardial infarction who underwent percutaneous coronary intervention were analyzed. After an intervention period of three months, the maximal oxygen uptake and metabolic equivalent of task were 5.53 ± 0.12 and 19.32 ± 0.17, respectively, in the intervention group, and 4.15 ± 0.13 and 16.52 ± 0.18, respectively, in the control group. After three months of intervention, there were significant differences between the two groups in all observed indicators (*p* < 0.05), except for low-density lipoprotein and the incidence of major adverse cardiovascular events (*p* > 0.05). The use of a 5G Internet of Things platform cardiac rehabilitation model effectively improved outcomes in patients with acute myocardial infarction who underwent percutaneous coronary intervention. **Trials registry**: The study protocol was registered at Chinese Clinical Trials Registry (ChiCTR), first trial registration 07/08/2023, identification number ChiCTR2300074435.

## Introduction

Cardiovascular disease (CVD) is the leading cause of death worldwide^1,2^. Acute myocardial infarction (AMI) is defined as myocardial necrosis induced by acute, persistent ischemia and hypoxia in the coronary arteries^3^. Although the prevalence of AMI has decreased recently owing to changes in the demographic risk factors and appropriate management of patients in the acute and post-acute phases of the index event^4^, AMI remains one of the most important diseases threatening human life and health^5^. Exercise-based cardiac rehabilitation (CR) is considered an effective intervention for improving heart function^6^, enhancing physical fitness^7^ and optimizing mental health. Research has shown that CR can reduce the rates of cardiovascular mortality, secondary events, and hospitalization^8,9^. However, center-based CR (CBCR) requires daily travel to a hospital or center for rehabilitation activities and accompaniment during the round trip, which is costly and time-consuming^10,11^. A previous survey also found that transportation problems were the number one problem influencing traditional CR participation^12^. These issues of transportation, time, and expense contribute to a low CR participation rate of approximately 10%–30%^13–15^. Some researchers recommend home-based CR (HBCR) as an alternative to CBCR. Research has shown that HBCR programs are feasible and effective. The risk of major adverse cardiovascular events (MACE) during HBCR seems very low^16^. However, there is an urgent need for improvement of HBCR due to the lack of monitoring equipment with objective indicators and timely transmission of data to doctors to obtain a personalized rehabilitation scheme. With the development of communication technology and the Internet of Things (IOT) technology, patients can use portable monitoring devices to perform rehabilitation exercises at home. This rehabilitation mode is called cardiac telerehabilitation (CTR)^17^, which to some extent decreases the time required for travel between the patient’s residence and the rehabilitation facility, and also reduces the monetary cost. However, the use of 3G and 4G transmission information technology for CR involves severe audio and video lag and delay^18^, which seriously affects the patient’s experience and satisfaction. As a result, although participation in CTR has improved, it is still not ideal. The coronavirus disease 2019 pandemic initiated renewed thoughts on the importance of non-face-to-face CR in intensely infectious disease epidemics^19,20^. In the present study, we used the latest 5G information transmission technology, which has low latency and enables synchronous transmission of audio and video. Furthermore, using the IOT technology, it is possible to synchronously transmit vital signs data obtained during rehabilitation sessions to the control center. This permits observation of the effects of remotely guided Ba Duan Jin exercise rehabilitation on physiological and psychological indicators during CR. We hypothesized that compared with CBCR, CTR using 5G information transmission technology and IOT technology would better improve patients’ cardiopulmonary ability, improve patients’ cardiovascular risk factors, reduce the levels of psychological anxiety and depression, significantly improve the CTR participation rate, and reduce the incidence of MACE.

## Methods

### Study design

This study was a single-blind, randomized, controlled trial. The study was approved by the Ethics Committee of the Second Affiliated Hospital, Guizhou University of Traditional Chinese Medicine (KYW2022007). The study conformed to the provisions of the Declaration of Helsinki, and was conducted at cardiology units in the Second Affiliated Hospital, Guizhou University of Traditional Chinese Medicine, Guiyang, China, from April 2022 to March 2023. Eligible participants were randomized to either the intervention group or control group using sequentially numbered sealed opaque envelopes. All randomization procedures were performed by a researcher who was not involved in patient recruitment, exercise training, or outcome evaluation. Given that the participants were recruited continuously as the trial progressed rather than at a single point in time, a permuted-block randomization method was used to ensure equal sample sizes between groups over time^21^. When eight individuals were successfully recruited, the randomization procedure (i.e., block size = eight) was conducted with an allocation ratio of 1:1 (i.e., four participants were assigned to the intervention group and four were assigned to the control group). The outcome variables were measured at baseline and after three months of intervention. Single-blinding was maintained as research assistants performing the data collection had no access to information about the group allocations.

### Sample size

The sample size was estimated using the following formula for two-group comparisons with a random design:

$$\mathrm{NI}=\mathrm{N}2=2\left[\frac{({\mathrm{Z}}_{\mathrm{\alpha }}+{\mathrm{Z}}_{\upbeta })\mathrm{ S}}{\updelta }\right]2$$

where Z_α_: the corresponding Z value for type I error α; Z_β_: the corresponding Z value for type II error β; $\mathrm{S}$: standard deviation; and $\updelta$: permissible error.

The unilateral test was adopted with α = 0.05, β = 0.20, Z_α_ = 1.96, and Z_β_ = 1.06. The total cholesterol, triglyceride, and low-density lipoprotein cholesterol were the primary outcome indicators. $\mathrm{S}=$ 0.26 and $\updelta$=0.18 based on a review of the related literature^22^. Therefore, the calculated result was N1 = N2 = 20. Assuming an attrition rate of 20%, we needed to recruit 24 patients in each group.

### Participants

During the study, patients were treated with medications and underwent PCI in the hospital for AMI and were followed clinically at regular intervals after discharge. Participants were recruited through study promotional posters and hospital staff referrals. Researchers screened the participants’ medical records and conducted interviews to determine the eligibility of individuals who expressed interest in participation. Inclusion criteria: age 60–75 years; ability to use electronic equipment; ability to perform self-care, with no physical activity impairment; left ventricular ejection fraction ≥ 40%; signed informed consent provided; and willing and able to attend the complete study program without assistance. Patients were ineligible if they met any of the following exclusion criteria at screening: site researchers considered that the patient was unable to complete the study and/or attend the follow-up visits; regular practice of Ba Duan Jin, i.e., more than three sessions per week; concurrent participation in any other clinical trials; and concurrent other pathologies or malignant tumors (e.g., severe valvular disease, New York Heart Association Class IV, heart failure, severe aortic incompetence, cancer, and end-stage renal or liver disease).

### Interventions

#### Usual treatment and care

Patients received routine medical and nursing care from community-based physicians and cardiologists after discharge from the hospital. Conventional treatment consisted of the postoperative administration of 100 mg aspirin (Bayer Leverkusen, Germany) once daily for long-term maintenance. Oral ticagrelor (AstraZeneca, Wilmington, DE) 90 mg twice daily was prescribed for one year. Other medications (statins, angiotensin-converting enzyme inhibitors, angiotensin II receptor blockers, calcium channel blockers, and β-receptor blockers) were given as needed according to the patient’s condition. Routine care comprised post-PCI patient health education and medication management information provided by ward nurses prior to discharge, and regular follow-ups. The routine medical care was in accordance with the guidelines for cardiovascular rehabilitation and secondary prevention in China 2018 simplified edition^23^.

#### Control group

Patients assigned to the control group received traditional in-hospital CR training combined with the traditional Chinese exercise Ba Duan Jin. The specific rehabilitation training was as follows: respiratory training for 10 min; aerobic exercise for 15 min; resistance exercise comprising simple dumbbell exercises for 10 min; Ba Duan Jin exercises for 10 min (see the appendix for the specific movements in the Ba Duan Jin, repeat each of the 8 segments three times); and balance and flexibility exercise relaxation training for 10 min. Patients were encouraged to visit the hospital three times a week for 50–60 min of exercise each visit. The age-predicted target heart rate (220 minus the patient’s age in years) was used as a measure of intensity regarding the aerobic portion of the exercise training regimen^24^. For all participants, the selected target for the aerobic exercise training portion of the program was 60% of the age-predicted target heart rate. The general rating of perceived exertion^25^ was also used to evaluate the exercise intensity, and the 11–14 grades were used as the exercise intensity index.

#### Intervention group

In addition to the exercise protocol, the intervention group used a wearable smart device and the 5G IOT CR intelligence platform for remote management of CR. CR was performed three times a week for 50–60 min each session. The total number of interventions was 36 sessions.

**Figure 1:**
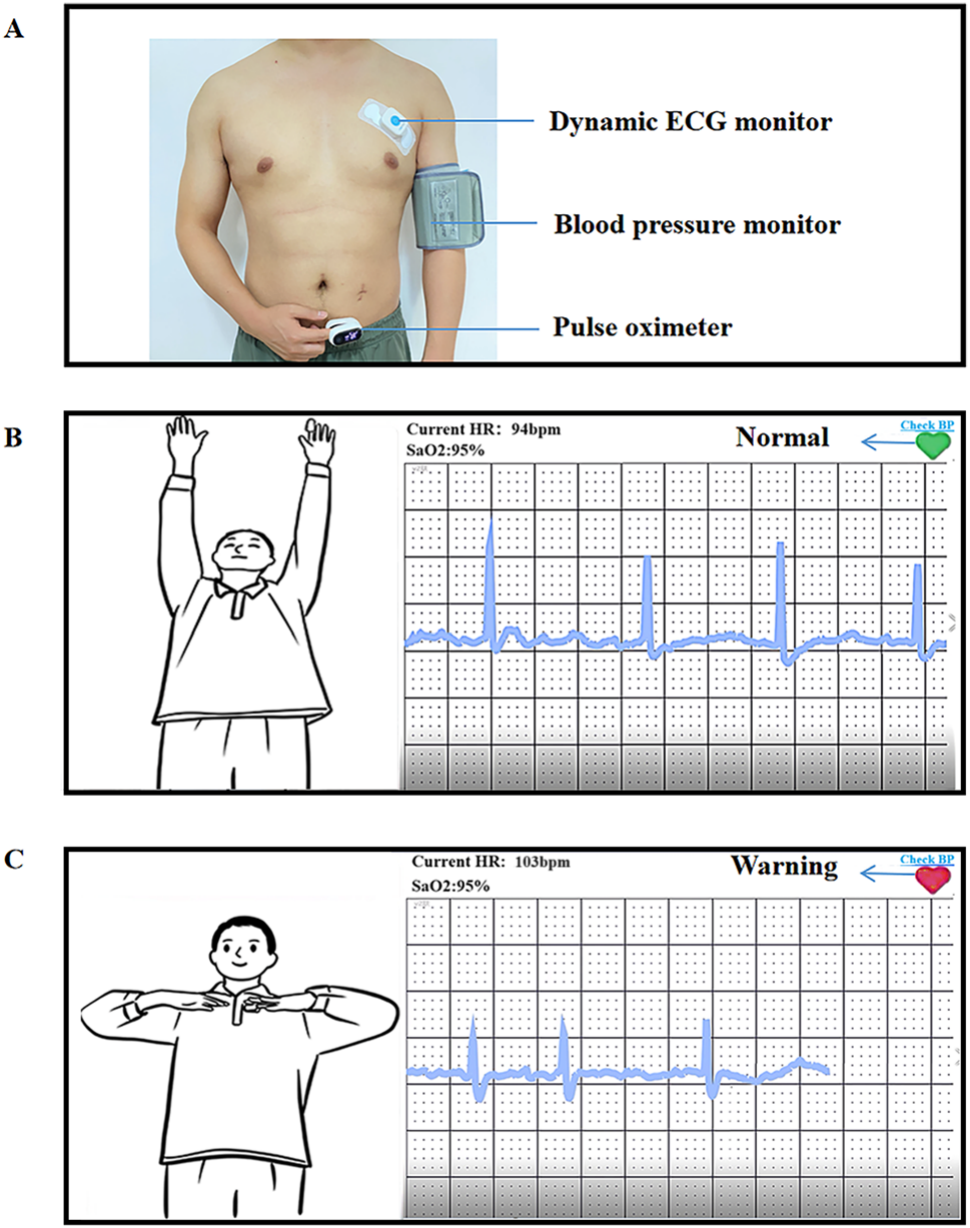
Portable cardiac rehabilitation equipment.

**Figure 2:**
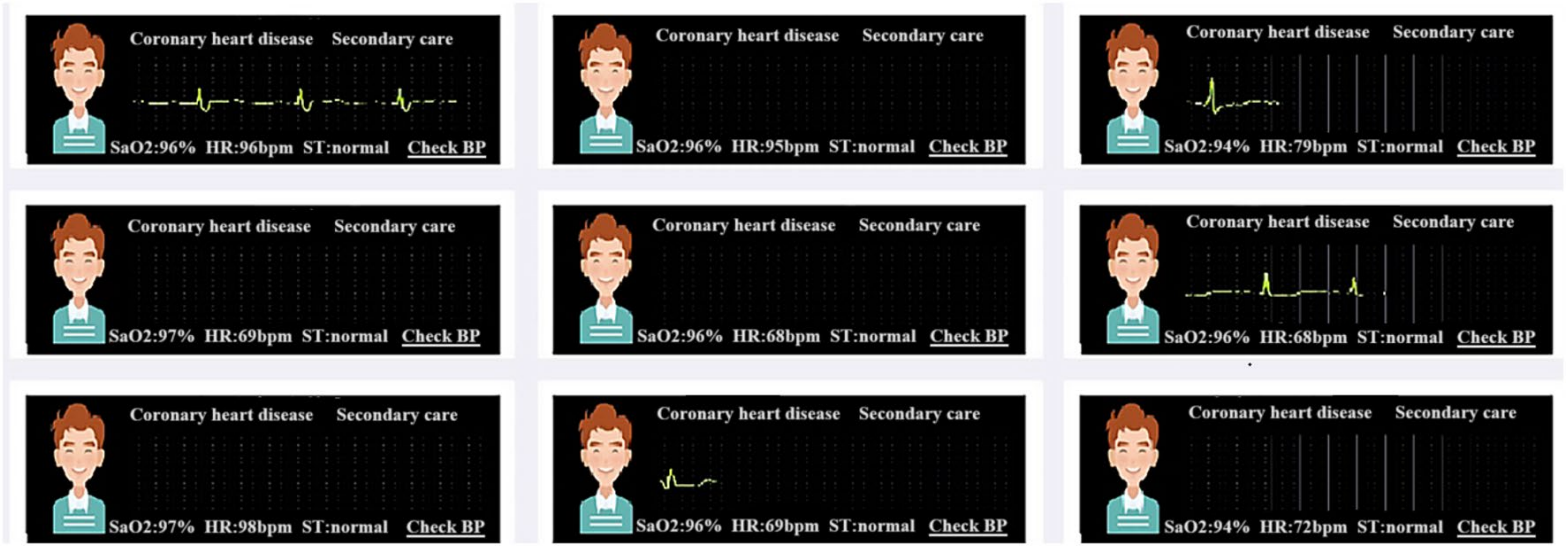
The operation of the 5G Internet of Things cardiac rehabilitation platform.

Researchers verified whether the patients’ mobile phones were 5G-enabled. If not, the research group equipped the patients with 5G-enabled mobile phones and installed the Healthy Life Cycle application (app) before the patients were discharged. Patients also received wearable smart remote monitoring devices that were connected to the Healthy Life Cycle app via Bluetooth (pulse oximeter, model: A0J-70C, Shenzhen City, China; dynamic electrocardiogram (ECG) monitor, model: ECG-P01, Hangzhou City, China; arm-type electronic blood pressure monitor, model: B65T, Shenzhen City, China) (Figs. 1 and 2).

**Figure 3:**
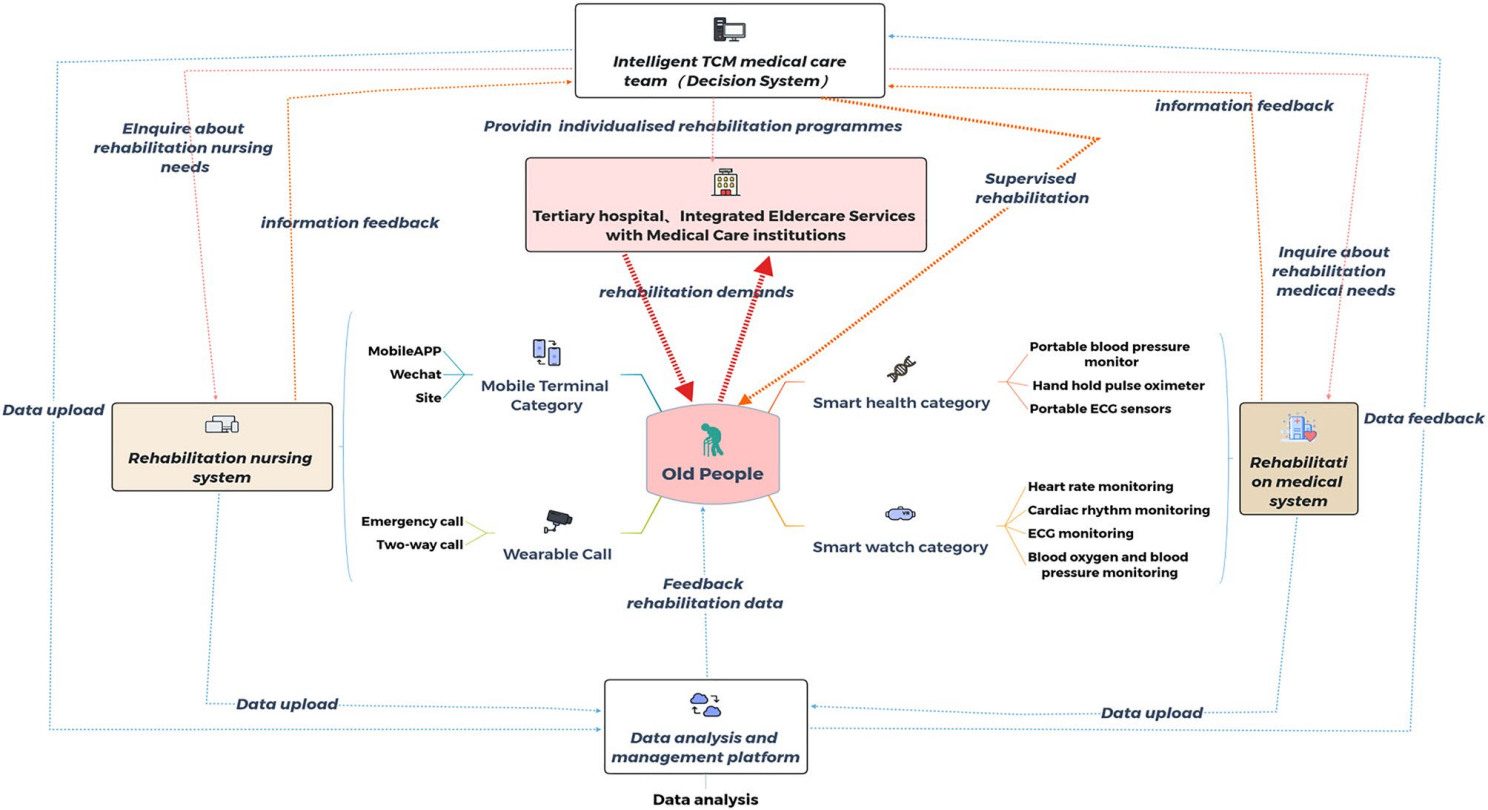
5G + IOT smart CR service architecture.

Patients were assisted by researchers and asked to log in to the Healthy Life Cycle app, click on “Rehabilitation Center” to access the online program, and wear the wearable device to monitor their vital signs and upload the data to the app (Fig. 3). The online program was open twice daily on weekdays at 9:00 a.m. and 14:30 p.m. Patients could choose to attend either session.

The rehabilitation exercise class was the same as that for the control group. Vital signs were recorded after each exercise, and the researchers ensured there were no abnormalities before the patients moved on to the next part of the exercise program. Patients could learn about their training and vital signs through the app, and the rehabilitator could adjust the exercise training pattern, intensity, and time for the patient on the basis of changes in the vital signs during exercise, to achieve personalized rehabilitation.

A psychologist was assigned to this study to provide psychological counseling to patients once a month to ensure their psychological well-being.

### Outcomes

The primary outcomes comprised changes in the cardiopulmonary exercise capacity, physiological indicators, and psychological indicators. Secondary outcomes were the patients’ exercise compliance, adverse cardiovascular events, and patient satisfaction with the rehabilitation care during the intervention. Cardiopulmonary exercise capacity, physiological indicators, and psychological indicators were measured before the intervention (baseline) and after three months of intervention. The patients’ exercise compliance, adverse cardiovascular events, and satisfaction with rehabilitation care during the intervention were investigated after three months of the intervention.

Cardiorespiratory capacity was measured using a cyclergometer cardiopulmonary exercise testing device (CS-200 Ergo-Spiro; Schiller, Switzerland) in accordance with the relevant cardiorespiratory exercise testing standards^26^. The maximal oxygen uptake (VO_2_max) and metabolic equivalent of task (MET) were used to evaluate the patients’ cardiac functional status and exercise capacity, respectively. Physiological indicators comprised high-density lipoprotein cholesterol, low-density lipoprotein cholesterol, triglycerides, total cholesterol, and body mass index. Psychological indicators were assessed using the Patient Health Questionnaire-9 (PHQ-9) scale^27^ and the Generalized Anxiety Disorder-7 (GAD-7) scale^28^.

Adherence to rehabilitation exercises and satisfaction with rehabilitation care were assessed by self-administered questionnaires. MACE, namely recurrent myocardial infarction, malignant arrhythmia, heart failure, and angina pectoris, were recorded during the intervention in both groups.

### Statistical analysis

Statistical analyses were performed using SPSS version 25.0 (IBM Corp., Armonk, NY). Data were reported as mean ± SD for continuous variables, and as counts and percentages for categorical variables. The two independent samples t-test was used for comparisons between groups. An analysis of covariance model was used to estimate differences in least square (LS) means with treatment (intervention versus control), and baseline values for the assessed variables as covariates. The LS means and between-group differences in LS means at three months were calculated for each variable along with the standard errors, 95% confidence intervals, and *p* values. The χ^2^ test was used for comparisons of categorical variables between groups. All reported *p* values were two-tailed, and *p* < 0.05 was considered to indicated statistical significance.

## Results

### Basic characteristics of the participants

**Figure 4:**
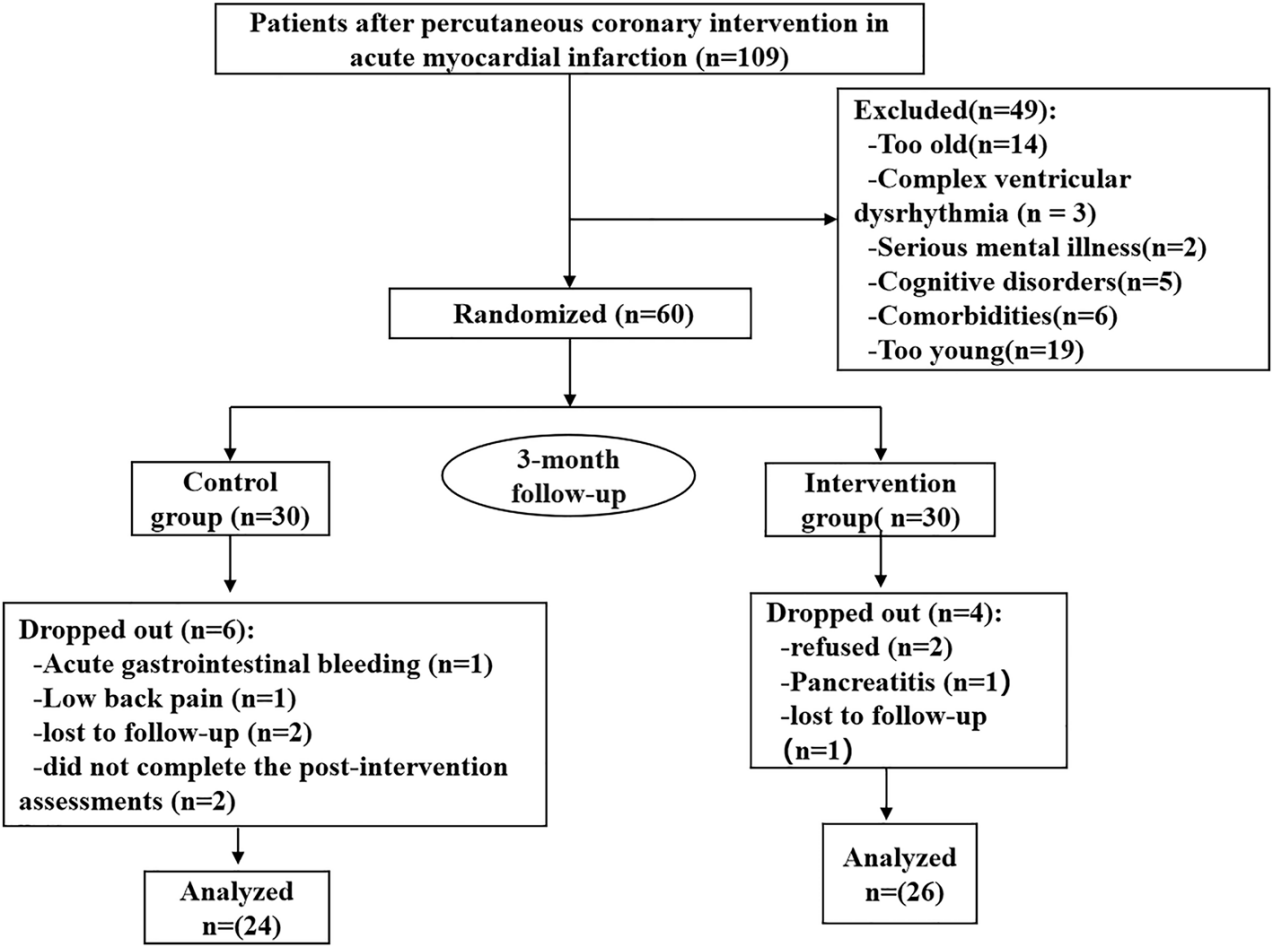
Flowchart of participant inclusion and exclusion.

We enrolled 109 patients with AMI who underwent PCI and agreed to participate in the study between April 2022 and March 2023. The researchers screened the patients using the inclusion and exclusion criteria and enrolled 60 patients; 30 each in the control and intervention groups. Ten participants left the study during the intervention. In the control group, one person developed acute gastrointestinal bleeding, one developed low back pain, two were lost to follow-up, and two refused to continue to participate in the study. Two people in the intervention group refused to continue to participate in the study, one developed pancreatitis, and one was lost to follow-up. The overall participation rate of the two groups was 83.3% (50/60). There were 24 participants in the control group and 26 in the intervention group (Fig. 4). The mean age of the overall study population was 66.28 ± 4.01 years and the overall number of female participants was 26 (52.0%). There were no significant differences between the groups in general data (all *p* > 0.05) (Table 1).

**Table 1:** Baseline characteristics in the intervention and control groups.

Characteristic	Control group (n = 24)	Intervention group (n = 26)	t	*p value*
Sex, n (%)			1.51	0.21
Males	12 (50.00)	12 (46.15)		
Females	12 (50.00)	14 (53.85)		
Age, (years)	66.58 ± 4.41	66.00 ± 3.75	1.01	0.32
Education level			1.00	0.13
Below junior high school, n (%)	16 (66.67)	19 (73.08)		
High school and above, n (%)	8 (33.33)	7 (26.92)		
Smokers, n (%)	13 (54.17)	8 (30.77)	1.51	0.21
Drinkers, n (%)	8 (33.33)	10 (38.46)	2.14	0.14
Hypertension, n (%)	17 (70.83)	18 (69.23)	1.68	0.19
Diabetes, n (%)	8 (33.33)	8 (30.77)	0.37	0.54
Hyperlipidaemia, n (%)	10 (41.67)	13 (50.00)	0.12	0.72
METS	3.62 ± 0.53	3.70 ± 0.52	1.39	0.17
VO_2 _max(ml/min/kg)	15.84 ± 1.83	15.59 ± 1.82	− 0.49	0.62
LDL-C, (mmol/L)	2.65 ± 0.53	2.64 ± 0.63	− 1.17	0.25
HDL-C, (mmol/L)	1.09 ± 0.38	1.00 ± 0.20	− 1.45	0.15
TCH (mmol/L)	4.34 ± 0.78	4.44 ± 0.64	0.33	0.73
TG, (mmol/L)	1.86 ± 0.47	1.87 ± 0.31	0.14	0.88
BMI (kg/m^2^)	22.68 ± 3.42	23.48 ± 2.80	1.23	0.22
GAD-7 (score)	5.75 ± 1.80	6.62 ± 2.21	2.04	0.05
PHQ-9 (score)	6.63 ± 2.01	6.62 ± 2.06	0.33	0.74

Data are presented as mean ± SD unless noted otherwise.

*METS* metabolic equivalent, *VO*_*2*_*max* maximal oxygen uptake, *LDL-C* low-density lipoprotein cholesterol, *HDL-C* high-density lipoprotein cholesterol, *TCH* total cholesterol, *TG* triglycerides, *BMI* body mass index.

### Comparison of motor ability between the two groups before and after intervention

At baseline, there were no differences between the two groups in the MET and VO_2_max. After three months of intervention, the MET and VO_2_max had improved in both groups, but these improvements were significantly greater in the intervention group than the control group. The difference between the two groups was statistically significant (*p* = 0.001) (Table 2).

**Table 2:** Comparison of exercise capacity between the two groups.

	Control group (n = 24)	Intervention group (n = 26)	Group difference (intervention-control, 95% CI)	t	*p value*
METS	4.15 ± 0.13*	5.53 ± 0.12*	1.39 (1.03–1.74)	7.89	0.001
VO_2 _max (ml/min/kg)	16.52 ± 0.18*	19.32 ± 0.17*	2.79 (2.29–3.29)	11.14	0.001

Data are presented as mean ± SD. Comparison with pre-intervention, *p < 0.05.

### Comparison of physiological indicators between the two groups before and after the intervention

After three months of intervention, there were statistically significant differences in HDL-C, TC, TG, and BMI between the two groups (*p* < 0.05), and there were no differences in LDL-C between the two groups (*p* > 0.05) (Table 3).

**Table 3:** Comparison of physiological indicators between the two groups before and after the intervention.

	Control group (n = 24)	Intervention group (n = 26)	Group difference (intervention-control, 95% CI)	t	*p value*
LDL-C, (mmol/L)	2.52 ± 0.07	2.42 ± 0.06	− 0.10 (− 0.28 to 0.09)	− 1.07	0.29
HDL-C, (mmol/L)	1.11 ± 0.02	1.17 ± 0.02	0.05 (0.01–0.10)	2.22	0.03
TCH, (mmol/L)	4.28 ± 0.04	3.85 ± 0.04	− 0.42 (− 0.54 to − 0.30)	− 7.01	0.001
TG, (mmol/L)	1.69 ± 0.04	1.55 ± 0.04	− 0.14 (− 0.25 to − 0.04)	− 2.69	0.01
BMI (kg/m^2^)	22.76 ± 0.14	22.29 ± 0.14	− 0.47 (− 0.87 to − 0.08)	− 2.39	0.02

Data are presented as mean ± SD.

### Comparison of psychological indicators between the two groups before and after the intervention

At baseline, there were no differences between the groups in the GAD-7 and PHQ-9 scores. After three months of intervention, the GAD-7 and PHQ-9 scores had decreased in both groups, but the decrease was significantly greater in the intervention group than the control group for the GAD-7 (*p* < 0.05) and PHQ-9 (*p* < 0.05) (Table 4).

**Table 4:** Comparison of psychological indicators between the two groups before and after the intervention.

	Control group (n = 24)	Intervention group (n = 26)	Group difference (intervention-control, 95% CI)	t	*p value*
GAD-7	4.79 ± 0.39	3.69 ± 0.37	− 1.10 (− 2.17 to − 0.03)	− 2.06	0.045
PHQ-9	4.89 ± 0.38	3.68 ± 0.36	− 1.20 (− 2.26 to − 0.14)	− 2.28	0.027

Data are presented as mean ± SD.

### Comparison of rehabilitation compliance between the two groups before and after the intervention

The overall rehabilitation compliance rate was significantly higher in the intervention group (80.8%) than the control group (29.2%) (*p* < 0.03) (Table 5).

**Table 5:** Comparison of rehabilitation compliance between two groups of patients n (%).

Group	Full compliance	Partial compliance	Non-compliance	Total adherence rate
Control group (n = 24)	3 (12.50)	4 (16.67)	17 (70.83)	7 (29.17)
Intervention group (n = 26)	9 (34.62)	12 (46.15)	5 (19.23)	21 (80.77)
χ^2^				10.47
*p value*				0.03

### Comparison of the incidence of MACE in the two groups

After three months of intervention, the incidence of MACE did not differ between the groups (*p* > 0.05), and the use of CTR did not increase the risk of MACE (Table 6).

**Table 6:** Comparison of the incidence of MACE in the two groups (n (%).

Group	Recurrent myocardial infarction	Malignant arrhythmia	Heart failure	Angina
Control group (n = 24)	1 (4.17)	3 (12.50)	2 (8.33)	3 (12.50)
Intervention group (n = 26)	1 (3.84)	2 (7.69)	1 (3.84)	2 (7.69)
χ^2^	0.05	2.81	0.10	0.31
*p value*	0.83	0.10	0.76	0.56

### Comparison of post-intervention satisfaction with rehabilitation care between the two groups

The overall score for satisfaction with the rehabilitation care was significantly higher in the intervention group (100%) than the control group (83.4%) (*p* < 0.01) (Table 7).

**Table 7:** Comparison of satisfaction with post-intervention rehabilitation care between the two groups n (%).

Group	Dissatisfaction	General satisfaction	Very satisfied	Total satisfaction
Control group (n = 24)	4 (16.67)	10 (41.67)	10 (41.67)	20 (83.33)
Intervention group (n = 26)	0 (0.00)	11 (42.31)	15 (57.69)	26 (100.00)
χ^2^				8.00
*p value*				0.01

## Discussion

PCI involves coronary balloon dilation and stenting to restore local coronary arteries to their normal diameter. However, PCI does not alter the underlying causes that lead to the development of coronary ischemia. Following myocardial infarction and PCI, there is a high incidence of significant adverse cardiac events^29^ and high rates of patient readmission and mortality^30^. CR is important to improve cardiac function and reduce the incidence of MACE in patients with CVD.

CR comprises exercise rehabilitation, diet, and health education. Exercise is the most important component of CR^31^, and all types of exercise improve cardiac function in patients with CVD^32,33^. Ba Duan Jin is a traditional Chinese sport that is popular in China^34^. Ba Duan Jin is easy to incorporate in the implementation and guidance of distant CR exercise. Numerous studies have shown that routine practice of Ba Duan Jin improves overall health^35,36^ and can be used for CR. In the present study, we used the MET and VO_2_max as indicators of exercise capacity for CR. At the end of the intervention, the MET and VO_2_max were both higher in the intervention group compared with the control group (*p* < 0.001). Furthermore, the degree of improvement in exercise capacity was greater in the intervention group than that in the control group. Research has shown that the peak oxygen consumption reached at the end of CR is closely related to the long-term survival rate of patients with coronary heart disease^37^.

The present study also monitored indicators closely related to the development of atherosclerosis, including low-density lipoprotein cholesterol, total cholesterol, and triglycerides. The levels of all three indicators were lower in the intervention group than the control group (all *p* < 0.05), while the level of high-density lipoprotein, which is a protective factor against atherosclerosis, was higher in the intervention group than the control group (*p* < 0.05). The control group also showed improved cardiac function and increased aerobic capacity, consistent with previous studies of CBCR^38,39^. CR with long-term follow-up improves the lipoprotein particle profile^40^. The present results proved that Ba Duan Jin was effective for CR^35^. The indicators of CR in the intervention group were better than those in the control group, which may be attributed to the high participation rate and good compliance (Table 5).

The intelligent wearable devices used for CR in the present study were pulse oximeters, dynamic ECG monitors, and arm-type electronic blood pressure monitors (Fig. 1A). These devices were used to monitor blood oxygen saturation, ECG (heart rate and rhythm), and blood pressure, respectively, and the data were transmitted to the data center (Fig. 1B). These devices reported an alarm when the patient’s heart rate exceeded an alert level or when the patient developed severe arrhythmia. The alarm was transmitted to both the patient and their doctor (Fig. 1C). The physician immediately told the patient to reduce the intensity of the training or to suspend training to prevent a MACE. Adjustments to the exercise in CBCR are made mainly on the basis of the patient’s self-perception during exercise. When patients experience role reinforcement, they will exaggerate the discomfort to a certain extent, leading to misjudgment by the doctor and a decrease in the rehabilitation effect due to insufficient exercise. The present study combined objective indicators with the patients’ subjective feelings, which was more scientific compared with subjective assessment only. Because the devices were equipped with alarms, patients felt much more secure during the rehabilitation process compared with performing rehabilitation exercises without a timely warning. This reduced the subjective reduction in exercise volume and intensity by patients, which enhanced the effect of the CR. Additionally, the devices can be used to monitor and remind patients to attend rehabilitation training on time through the data center (Fig. 2). A previous study showed that CTR improves adherence to CR and enhances the effectiveness of rehabilitation through WeChat reminders^41^. The compliance of the present study was 80.8%, which was lower than that of a home rehabilitation study using a wrist heart rate monitor^42^. The possible reason for this discrepancy was that the present study included older adults, some of whom were less receptive to the use of new technologies and methods than young people. This suggests that people's preferences should be considered when designing research to ensure user engagement^43^. For patients with poor digital literacy or limited digital access, available family members or close friends can be used to help improve the use of digital devices, so that patients can better participate in self-monitoring and behavior adjustment^44^. As a result of the technological support provided by the researchers, patient compliance in the present study was higher than that in previous studies of CTR. We hypothesized that the dual effect of supervisory reminders issued by the data center and the sense of security given to patients by the early warning system produced these positive results.

Physiology and psychology influence each other in disease development and progression. Anxiety is prevalent in patients with CVD^45,46^ and causes vasoconstriction^47^ and worsens coronary artery ischemia. Studies have shown that anxiety and depression are closely related to the prognosis of heart disease^48^. In the present study, we evaluated physiological and psychological indicators of anxiety and depression. The results showed that the levels of anxiety and depression significantly decreased in the intervention and control groups (Table 4). The present results were consistent with the conclusions of Kraal et al.^49^, proving that remote CR and family-based CR are also effective in improving patients' mental health.

Communication technology allows patients in areas located away from major cities to participate in CR and receive personalized exercise guidance through 5G and IOT technology. This approach enables the coordination of high-quality medical resources.

In conclusion, during CR using ultra-low latency 5G technology combined with wearable smart devices, patients were able to observe improvements in their physiological indicators and experienced decreases in depression and anxiety. The reduction of anxiety and fear of exercise associated with close monitoring motivated patients to exercise and achieve the maximum amount of exercise within the safe range. This led to improved physiological indicators and achieved the desired results. The test results were consistent with our hypothesis. Artificial intelligence has been gradually applied to CR^50^. In the future, artificial intelligence and deep learning will enhance the construction of risk prediction models for precise CR.

### Supplementary Information

Supplementary Information.

## Data Availability

The datasets generated and analyzed in the current study are not publicly available due to the identity information contained in the data. Deleting this information can obtain them from the corresponding author upon reasonable request.
