# Supplementary material for: Cardiac telerehabilitation under 5G internet of things monitoring: a randomized pilot study
Source: Sci Rep. 2023 Nov 2;13:18886. doi: 10.1038/s41598-023-46175-z (PMC10622509; doi:10.1038/s41598-023-46175-z)

Full title of the article: Cardiac telerehabilitation under 5G internet of things monitoring: a randomized pilot study

Xiaojie Li[1†],PhD;Lvheng Zhao[1†],MS;Tao Xu[2†], MD;Guofeng Shi[1†],MS; Jie Li[1], ,MS;Wei Shuai[3], MPA; Yanqun Yang[2], MS;Yang Yang[2],MS; Weiyi Tian[4*],MD, PhD and Yixia Zhou[1*], PhD.

1.Nursing School of Guizhou University of Traditional Chinese Medicine, Guiyang, Guizhou, China

2. Department of Cardiovascular Internal Medicine, Second Affiliated Hospital, Guizhou University of Traditional Chinese Medicine, Guiyang, Guizhou, China

3.Second Affiliated Hospital, Guizhou University of Traditional Chinese Medicine, Guiyang, Guizhou, China

4. Guizhou University of Traditional Chinese Medicine, Guiyang, Guizhou, China

†These authors contributed equally to this work and share first authorship.

*Corresponding author: Weiyi Tian, MD, PhD, Guizhou University of Traditional Chinese Medicine, Guiyang, Guizhou, China 550025, China. Tel.: +86-13595040853E-mail: tianweiyi@gzy.edu.cn.

*Correspondence author: Yixia Zhou, PhD, Nursing School of Guizhou University of Traditional Chinese Medicine, Guiyang, Guizhou, China, 550025, China. Tel.: +86-15908515850, E-mail: zhouyixia2014@126.com

Form 1. Holding the Hands High with Palms Up to Regulate the Internal Organs

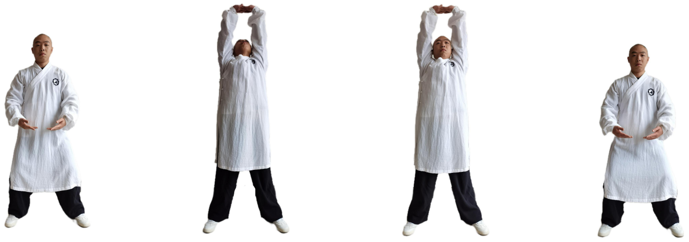

Form 2. Posing as an Archer Shooting Both Left- and Right-Handed

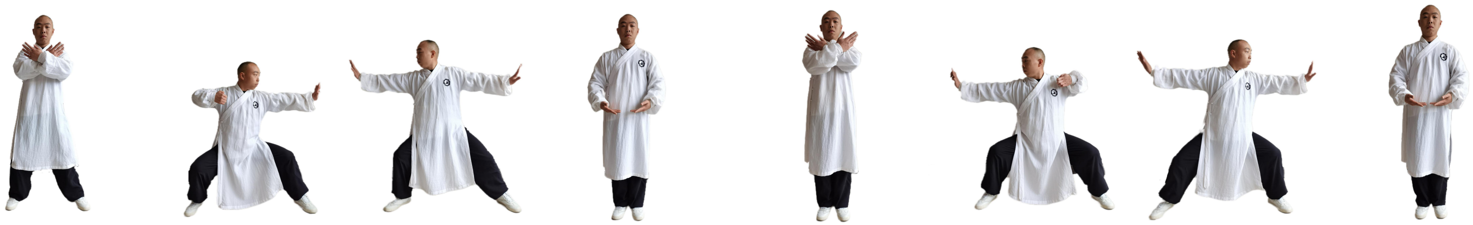

Form 3. Holding One Arm Aloft to Regulate the Functions of the Spleen and Stomach

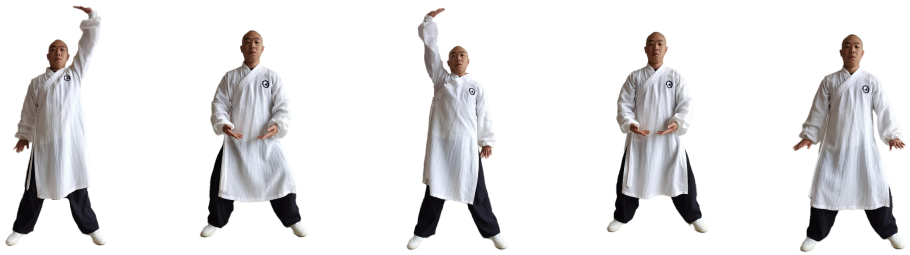

Form 4. Backwards to Prevent Sickness and Strain

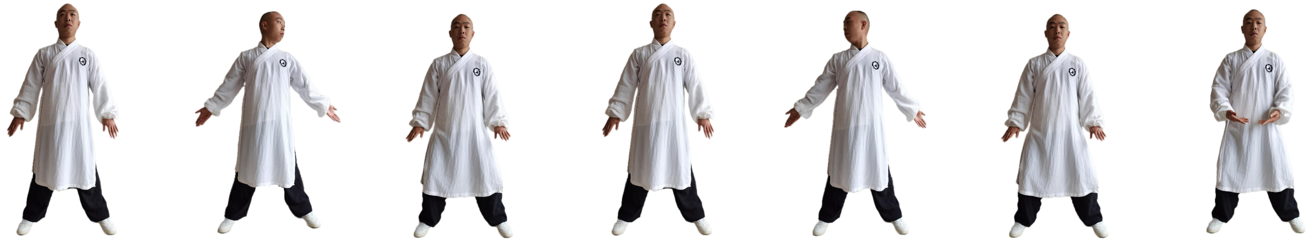

Form 5. Swinging the Head and Lowering the Body to Relieve Stress

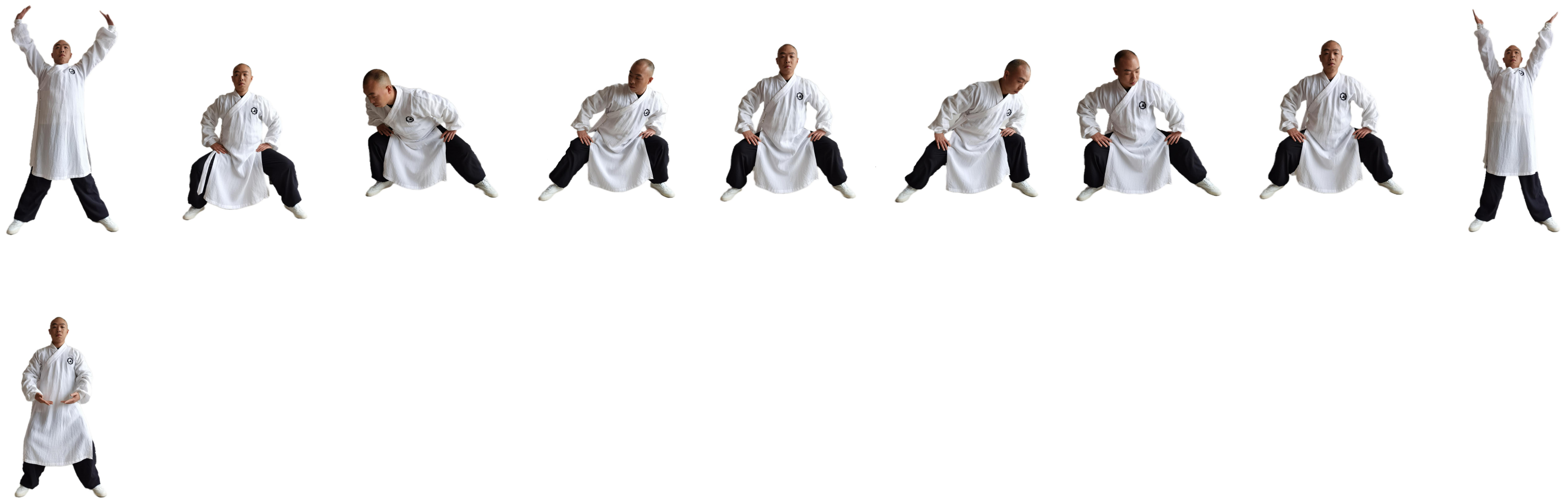

Form 6. Moving the Hands down the Back and Legs,and Touching the Feet to Strengthen the Kidneys

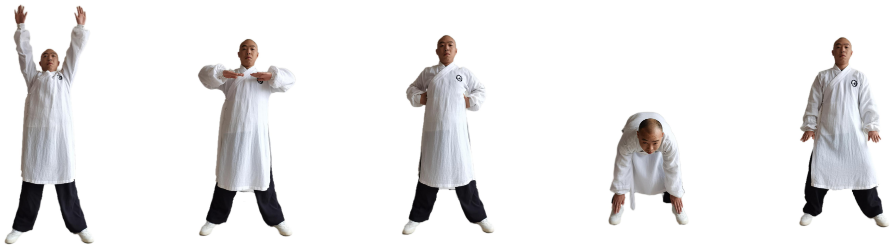

Form 7. Thrusting the Fists and Making the Eyes Glare to Enhance Strength

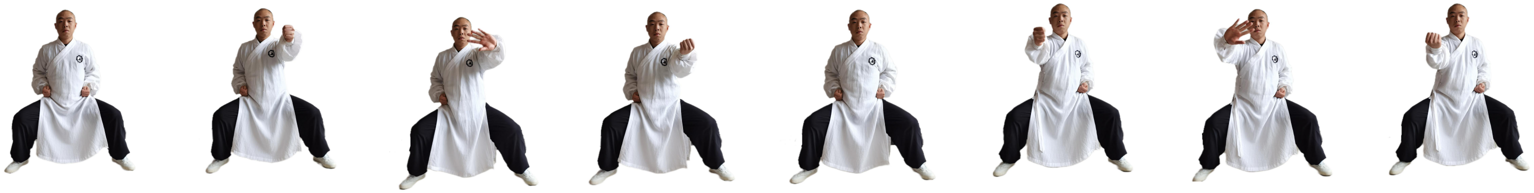

Form 8. Raising and Lowering the Heels to Cure Diseases

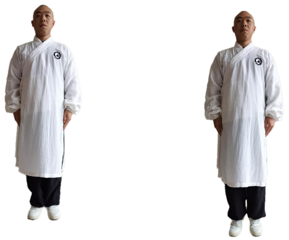

Supplement: Supplementary file 1 — Supplementary Information. [file 41598_2023_46175_MOESM1_ESM.pdf]
